# Global warming and the Summer Olympic and Paralympic games: a perspective from the Tokyo 2020 Games

**DOI:** 10.1265/ehpm.21-00024

**Published:** 2022-03-04

**Authors:** Lisa Yamasaki, Shuhei Nomura

**Affiliations:** 1 Department of Global Health Policy, Graduate School of Medicine, The University of Tokyo, Tokyo, Japan; 2 School of Medicine, Nagasaki University, Nagasaki, Japan; 3 Department of Health Policy and Management, School of Medicine, Keio University, Tokyo, Japan; 4 Tokyo Foundation for Policy Research, Tokyo, Japan

**Keywords:** Heat-related illness, Climate change, Mass gatherings, Environmental health, The summer Olympic and Paralympic games

## Abstract

The Tokyo 2020 Olympic and Paralympic Games provided a significant opportunity to consider global warming as an issue to be seriously addressed to run the safe and fair games in the era of climate change. As the global temperature continuously rises and extreme hot-weather events increase in frequency and intensity, the future summer Olympic and Paralympic games will need to deal with the heat by applying thorough and appropriate countermeasures. In the recent decades, many mitigation measures to protect athletes from heat have been rapidly discussed by the sports community, including countermeasures to hold games at times and places with moderate temperature and climatic risk assessments with Wet Bulb Globe Temperature (WBGT) during the games. However, the excessive heat conditions in the Tokyo 2020 Games affected not only athletes, but also all people concerned the events. While deliberate considerations by organizers had been given to mitigate extraordinary heat, the evaluations of these measures and epidemiological analyses of risk factors of patients must be further enhanced to develop efficient measures for the future. Therefore, we discussed the underlying climate-related problems of the summer Olympic and Paralympic Games in view of what we had experienced in the Tokyo 2020 Games. Facing with emerging global warming, future intervention against heat in the summer Olympic and Paralympic games will need to integrate systematic disease surveillance and evaluation of intervention with an effective combination with the approaches previously conducted. The Tokyo 2020 Games is a wake-up call to accelerate the public health measures towards the creeping global warming.

## Introduction

The issues to grow with the Summer Olympic and Paralympic games in the era of global warming have been discussed recently. [1, 2] The Tokyo 2020 Olympic and Paralympic Games brings us an opportunity to recognize the climate impact to deliver the safe and fair games for all people concerned in the event. Environmental heat stress is detrimental to the ‘once-in-a-lifetime’ performances for athletes, requiring them to perform their best while challenging the limit of human physiology. Not only athletes, in large-scale mass gatherings, such as the Olympics and Paralympic games, staff and spectators increase the potential of heat-related illness under the extreme hot climate. Patients of heat-related illness may suffer from subsequent complications and even fatal situation in some worse cases. [3] There is a crucial need to focus on how to organize the safe and fair Olympic and Paralympic games for athletes, staff, and spectators in the 21^st^ century with emerging global warming.

## Preparation against heat in sports events

The sports community has recently possessed the importance of mitigation measures of the heat effect on athletes especially for outside sports since competitive sports in a hot environment affect the various component of physical performance and may lead to outcome of the games. [4–6] For example, FIFA World Cup Qatar 2022 has explicitly addressed this issue: they have reconsidered the season of games from June to shift into November and December in 2022 in the moderate season and arranged for air-conditioned stadiums so that athletes and spectators are less affected by intense heat during the matches. [7] Besides considerations of the time and place to hold the games, climatical risk assessment during the games by using Wet Bulb Globe Temperature (WBGT) has been rapidly evolving and widely accepted. [8] Each of the international sports federations and sports communities sets the WBGT criteria to suspend the games which differs depending on characteristic of sports. [9] Medical countermeasures such as dedicated medical facilities and related educational materials are also useful. We need to turn these discussions of the heat protection measures into effective actions to encounter rapid progressing global warming.

## Athletes and heat in the Tokyo 2020 Games

The Tokyo 2020 Games were one of the most gruelling games in their histories due to the extremely high temperature and humidity in Japan. The game period hit the hottest time in Japanese summer when the burden of heat-related illness is the most prominent. [10] In the Tokyo Olympic Games, organizers have proactively contributed the heat countermeasures such as shifting venues from Tokyo, the host city, to Sapporo, the northeast prefecture of Japan where summer temperatures are moderate and scheduling the starting time of games early in the morning or evening to avoid midday sun for some sports. These measures were aimed to protect athletes from heat based on scientific consideration by the International Olympic Committee (IOC) Medical and Scientific Commission Adverse Weather Impact Expert Working Group (the IOC Working Group) [11]. However, despite the efforts by the organizers, intense heat had forced the competition schedule change just one day before the matches, for the tennis, marathon and soccer matches in the Olympic Games. The women’s soccer final was not only rescheduled, but also the stadium was changed under the influence of extreme hot temperature for the first time in the history of the Olympics. The sudden schedule change affected the athletes and may have made it difficult for them to perform in their best conditions. Moreover, one archer had fainted during the competition and three tennis players had retired midway through their respective matches due to the heat-related illness during the Tokyo Olympic game. [12] Although many elaborate precautions had been implemented to deal with heat before and during competitions, there is no refusing the fact that the extreme climate conditions had threatened the delivery of games in the Tokyo Olympic and Paralympic Games.

## Need to evaluate heat-related risks for non-athletic people

As for other relevant persons including staff and spectators, the Tokyo 2020 Games offered several unique heat protection measures. Alibaba, one of the largest sponsors of games, had provided onsite staff in Tokyo with wearable devices which keep track of signs of heatstroke, such as body temperature and heart rate. [13] Staff at increased risk of heat-related illness were automatically alerted and notified of prevention measures such as hydration based on the biometric data and the current environment index monitored by WBGT meters in the venues. Also, spectators were allowed to bring their own bottled drinks into the stadium which was not the case in the past Olympics and Paralympics for security reasons. Many other approaches including new digital solutions were adopted in the Tokyo 2020 Games. [14] Although it is important to measure the incidence of heat-related illness under population at risk in order to explore the stringent evidence of the countermeasures taken by the organizers, there is room for improvement in the evaluation of intervention effectiveness. In past documents of the Olympics and Paralympic games, the organizers have not disclosed the number and details of cases of heat-related illness during the games. In the Tokyo Olympics, they only reported “There is some minor heat illness, but not proper heat stroke, which is the most severe form of heat illness.” in the International Olympic Committee official website. [15] According to media information which is based on press conference of the Tokyo Organizing Committee of the Olympic and Paralympic Games, 59 athletes and 91 other relevant persons of heat-related illness were reported during 17 days of the Tokyo Olympics [16] out of total number of 11500 and 79000 [17] who may work for either outside or inside sports. With regard to the Tokyo Paralympics, 15 athletes and 17 other relevant persons were reported as heat-related cases [18] out of 4403 and 12000 [19] in the first 12 days of games. During the COVID-19 crisis, the Tokyo 2020 Games had been held without spectators except for some venues and had a great reduction of onsite staff, which may have resulted in avoiding serious heat-related issues related to the Tokyo 2020 Games in the general public.

## Future directions of the Olympic and Paralympic games in the era of global warming

While the future hosts of Olympics and Paralympics are picked more than 10 years before the games, the climate conditions make a rapid progress [20] and careful consideration are needed with the selection of host cities and seasons. In recent years, the summer Olympic and Paralympic games have been held in the hot summer season in the north hemisphere. As one study suggested, we will have only eight (1·5%) of 543 cities - outside of western Europe - as low risk category (<10% probability of ≥26°C WBGT) to run the safe games by 2085. [1] We will undoubtedly face this issue in the near future. Confronting this challenge will need solutions in a more planned and consistent way besides the previous concerned points such as considerations of time and place of games and strengthening of emergency care, which are basically well-designed for athletes. Looking forwards, we would suggest the organizers to activate the public health measures to prepare against heat as a real threat, not only for athletes, but also all people concerned such as staff and spectators in accordance with evaluation of these measures to identify trend in heat-related cases and underlying risks. This includes the epidemiological analyses of risk factors and exposure intensity of patients together with consideration of disease severity to accelerate more evidence-based and effective strategies for the future. In a mass gathering event like the Olympic and Paralympic games, many staff and spectators are involved and protection measures for them should not be neglected.

## Conclusion

Climate change is our reality. Transparent, inclusive, and practical strategies against heat should be shaped since severe weather conditions are becoming significant issues to all concerned in the Olympics and Paralympic games. Advancing systematic disease surveillance and evaluation systems across a sequence of the Olympic and Paralympic games would be helpful in learning from the past Olympic and Paralympic games. These approaches will help to handle the safe and fair Olympics and Paralympics in a time of global warming. We expect that the organizers will show leadership in combating global warming as a serious and pressing issue to be addressed in this century. The Tokyo 2020 Games is a wake-up call to change the conventional attitude towards dealing with the creeping global warming.
